# A review of the current policies and guidance regarding Apgar scoring and the detection of jaundice and cyanosis concerning Black, Asian and ethnic minority neonates

**DOI:** 10.1186/s12887-024-04692-4

**Published:** 2024-03-21

**Authors:** Amy Furness, Frankie Fair, Gina Higginbottom, Sam Oddie, Hora Soltani

**Affiliations:** 1 Sheffield Hallam University, Sheffield, UK; 2 University of Nottingham, Nottingham, UK; 3 Bradford Teaching Hospitals NHS Foundation Trust, Bradford, UK

**Keywords:** Infant, Newborn, Neonatal jaundice, APGAR score, Cyanosis, Ethnic and racial minorities, Ethnicity, Skin pigmentation, Policy, Skin pigmentation, Colour

## Abstract

**Background:**

Ethnic inequalities in maternal and neonatal health in the UK are well documented. Concerns exist regarding the use of skin colour in neonatal assessments. Healthcare professionals should be trained to recognise symptoms of diverse skin tones, and comprehensive, and inclusive guidance is necessary for the safe assessment of all infants. Disparities in healthcare provision have been emphasised during the COVID-19 pandemic, and additional research is needed to determine whether such policies adequately address ethnic minority neonates.

**Methods:**

A desktop search included searches of guidance produced for the United Kingdom (UK). Further searches of the Cochrane and World Health Organization (WHO) were used to identify any international guidance applicable in the UK context.

**Results:**

Several policies and one training resource used descriptors ‘pink,’ ‘pale,’ ‘pallor,’ and ‘blue’ about neonatal skin and mucous membrane colour. No policies provided specific guidance on how these colour descriptors may appear in neonates with different skin pigmentation. Only the NICE guidance and HEE e-learning resource acknowledged the challenges of assessing jaundice in infants with diverse skin tones, while another guideline noted differences in the accuracy of bilirubin measurements for the assessment of jaundice. Three policies and one training resource advised against relying on visual observation of skin colour when diagnosing neonatal conditions. The training resource included images of ethnic minority neonates, although most images included white infants.

**Conclusions:**

Inadequate consideration of ethnicity in UK policy and training perpetuates disparities, leading to inaccurate assessments. A review is needed for inclusivity in neonatal care, regardless of skin pigmentation.

**Supplementary Information:**

The online version contains supplementary material available at 10.1186/s12887-024-04692-4.

## Introduction

In the United Kingdom (UK), it is widely recognised that there are ethnic inequalities in maternal and neonatal health and care provision, with women from Black and Asian backgrounds having higher rates of maternal mortality [1], stillbirth and neonatal mortality than women of white ethnicity [2].

Health and care guidelines need to be comprehensive and inclusive to standardise practice; however, local settings vary considerably in their existing guidelines and practices [3]. All policies should be formulated to help reduce health inequalities and health disparities [4, 5] and, therefore, should consider the entire population.

Concerns have been raised regarding routine perinatal practices, such as Apgar scores, in which skin colour is a key element of neonatal examination. A much-debated question is whether neonates’ colour is assessed appropriately when scoring the Apgar [6] and whether this disadvantages infants with darker skin pigmentation. Guidelines should mitigate these concerns and describe effective and safe means of assessment for all infants. Concerns have also been voiced for other clinical assessments that use skin colour as an element of diagnosis. Moreover, visual inspection of a neonate’s skin colour is traditionally used to determine jaundice, which is often subjective and can be inaccurate, with skin pigmentation presenting as a confounding factor [7, 8]. Furthermore, diagnosing poor oxygenation in infants is difficult using visual assessment alone, and the diagnosis varies among clinicians, especially for infants with darker skin tones [9]. Due to the variations and unreliability of these assessments, clear national guidance is needed for healthcare professionals to assess all infants safely.

Textbooks and training have repeatedly been modelled on white skin in the UK and across the globe with little questioning [10, 11]. In a multiethnic society, the competence to recognise clinical symptoms in varied skin tones is crucial for providing holistic care and supporting healthcare professionals in their assessments. Most recently, gaps in healthcare professionals’ knowledge of dermatological assessment of different skin colours have been identified due to the limited diversity of educational resources and publications [12, 13].

The incidence of the COVID-19 pandemic highlighted the disparities in healthcare provision and supporting technology, with pulse oximeter readings taken in dark-skinned individuals being more likely to yield inaccurate readings [14, 15]. It is argued that aspects of racism in healthcare are structural and therefore embedded in our policies, laws and society [16]. Research to date has not yet determined whether Black, Asian or ethnic minority neonates are accurately described and considered in UK national policy.

## Methods

### Aim

This review aimed to examine current policies and guidance concerning the consideration of skin colour in black, Asian, and minority ethnic neonates through common assessments, such as Apgar scoring (including an assessment of oxygenation) and the detection of jaundice.

### Inclusion and exclusion criteria

The documents deemed suitable for inclusion encompassed clinical or practice guidelines, frameworks for practice (including draft frameworks), quality standards, good practice points, and pertinent educational resources.

Any policy or guideline addressing neonatal care, encompassing routine practices and specific practices such as Apgar scores, cyanosis, or jaundice, met the inclusion criteria.

Guidelines or policies looking at pharmacological practices were excluded.

### Search strategy

An informal review of relevant guidelines was conducted and encompassed searches of guidance produced by the British Association of Perinatal Medicine (BAPM), Institute of Health Visiting (iHV), National Institute for Health and Care Excellence (NICE), Neonatal Nurses Association (NNA), Office for Health Improvement and Disparities (OHID), Resuscitation Council UK, Royal College of Obstetricians and Gynaecologists (RCOG), Royal College of Paediatrics and Child Health (RCPCH) and the Royal College of Midwives (RCM). The focus of these searches was on guidance from the United Kingdom (UK). Additional exploration of the Cochrane Library database and the World Health Organization (WHO) aimed to identify any global guidance relevant to the UK. Example search strategies can be found in Additional file 1. Only the latest version of the guidance was incorporated, with a focus on documents published from 2010 onwards to guarantee contemporary relevance. DansEasy and Google were also searched for materials meeting the inclusion criteria. Furthermore, input from field experts and members of the project advisory committee was sought to identify any policies or guidance deemed pertinent for inclusion.

### Study selection

The retrieved guidance was independently screened by two researchers following the inclusion criteria. Any discrepancies were reviewed by a third researcher. Wherever practical, the sources referenced in the guidance concerning the application of assessments in Black and Asian neonates were also retrieved. Additionally, the supplementary materials included in the documents were examined and screened for their content.

### Critical appraisal

An evaluation of the guidelines using the AGREE II (Appraisal of Guidelines for Research & Evaluation II) instrument [17] was considered beyond the scope of this review. Therefore, an assessment was conducted concerning the relevance of the guidelines to skin colour and the impact of ethnicity on neonatal assessment.

### Analysis

Each guideline and publication were analysed for content about the utilisation of skin colour in diagnosing the aforementioned conditions. Two researchers identified keywords deemed applicable to the research question, and these were confirmed by the team. The identified keywords were used to search within the included documents, aiming to pinpoint relevant sentences and paragraphs in the text. The keywords used were: ‘Apgar’, ‘pale’, ‘blue’, ‘colour’, ‘color’, ‘cyanosis’, ‘hypox’, ‘oxy’, ‘red’, ‘yellow’, ‘pink’, ‘jaundice’, ‘Black’, ‘Asian’, ‘Cauc’, ‘ethnicity’, ‘pigment’, ‘skin’, ‘dark’, and ‘ethnic’. Quotes used for analysis can be found in Additional File 2.

### Data synthesis

A narrative synthesis of the findings was conducted, elucidating the application of practices and recommendations to Black, Asian, or ethnic minority neonates. This involved an inspection of the language utilised in guidance that detailed the observation of the skin (e.g., Apgar score, jaundice, and cyanosis).

## Results

We identified 18 guidelines, frameworks for practice, and quality standards designed for healthcare professionals (Table. 1). Additionally, learning tools created by one organisation were also identified (Table. 2). Among the 18 policies, guidelines, and training resources analysed, nine concentrated on the overall care of neonates, six guided the assessment of cyanosis or hypoxia, one addressed neonatal assessment using the Apgar score, and two focused on the assessment of jaundice. Out of the 18 policies, 15 were from the UK, and three were of international origin.

**Table 1:** Characteristics of the identified guidelines

No	Year of publication	Organisation	Title	Type of file
1	2022a	**British Association of Perinatal Medicine (BAPM)**	Postnatal care of the late preterm Infant	Draft Guideline [20]
2	2022b	**British Association of Perinatal Medicine (BAPM)**	Deterioration of the newborn	Draft Guideline [19]
3	2015	**British Association of Perinatal Medicine (BAPM)**	Newborn Early Warning Trigger and Track (NEWTT)	Framework for practice [18]
4	2022	**Institute of Health Visiting (iHV)**	Updated Good Practice Point – Babies who have neonatal jaundice	Good Practice point [34]
5	2010 Guideline (updated 2016) & 2014 quality standard.	**NICE** (National Collaborating Centre for Women and Children’s Health) & **RCOG** (Royal College of Obstetricians & Gynaecologists)	Jaundice in newborn babies under 28 days	Guideline [35, 38]
6	2019	**NICE**	Specialist neonatal respiratory care for babies born preterm [Full report]	Guideline [21]
7	2017	**NICE**	Intrapartum care for healthy women and babies	Guideline [30]
8	2021	**NICE and RCOG**	Postnatal care	Guideline [22]
9	No date a	**Neonatal Nurses Association (NNA)/University of Hertfordshire**	Assessment of the neonate	Resources for Nursing Practice [23]
10	No date b	**NNA/University of Hertfordshire**	Monitoring vital signs in the neonate.	Resources for Nursing Practice [36]
11	2021	**Office for Health Improvement and Disparities (OHID)**	Newborn infant examination	Guidance [24]
12	2011	**Resuscitation Council UK**	Air/oxygen blenders and pulse oximetry in resuscitation at birth	Quality standard [37]
13	2021	**Resuscitation Council UK**	Newborn resuscitation and support of transition of infants at birth Guidelines	Guideline [27]
14	2012	**Royal College of Midwives (RCM)**	Immediate care of the newborn	Guidelines [25]
15	2017	**Royal College of Nursing (RCN)**	Standards for assessing, measuring and monitoring vital signs in infants, children and young people	Guideline [26]
16	2022a	**World Health Organization (WHO)**	WHO recommendations on postnatal care of the mother and newborn	Guideline [33]
17	2022b	**WHO**	Early essential newborn care	Clinical practice guide [28]
18	2012	**WHO**	Basic newborn resuscitation	Guideline [45]

**Table 2:** Health Education England e-learning packages [29]

Module title	Topics included
**Avoiding Term Admissions Into Neonatal Units (ATA)**	Physiology of Jaundice. Term Newborn Babies at Risk of Jaundice First-Hour Care of the Term Newborn Infant Respiratory Distress
**NHS Newborn Infant Physical Examination (NIPE) Programme**	Screening and the Newborn and Infant physical examination (NIPE) Screening examination of the eyes Screening examination of the cardiovascular system Further information for NIPE practitioners
**Midwifery Identification, Stabilisation and Transfer of the Sick Newborn (MIST); Midwifery Identification, Stabilisation and Transfer of the Sick Newborn**	Colour (Anaemia and Cyanosis) Feeding and Abdominal Concerns Prematurity Hypoxia and Encephalopathy

### Routine care

#### General skin colour

Skin colour assessment as a component of routine care was addressed within 11 guidelines [18–28] and one training resource [29].

Guidelines propose that an assessment of skin colour within the first few hours of life should be utilised to evaluate the newborn’s adaptation to extrauterine life [19, 20, 22, 25, 27, 28], including for preterm infants [21]. For example, observations should include the following:

#### Discoloured peripheries and alternative locations

Additional guidelines highlighted that discoloured peripheries could signal a change in the infant’s condition, which may warrant closer observation, particularly during skin-to-skin contact.

Colour is a key element in the neonatal early warning trigger and track (NEWTT) chart [18]. . In the guidance, a point of concern was defined as an infant who appeared “*pale or blue or had an oxygen saturation <* 90”, while a colour of “*pink*” or oxygen saturation above 95% was considered normal [18]. According to the updated draft guidance, skin colour components that are *“very pale or blue”* are considered concerning, while *“pink or normal”* is not a cause for concern [20].

Various guidelines recognise that evaluating sufficient infant respiration involves considering multiple components, with skin colouring being just one of them [26]. In addition to assessing skin colour, the NNA/University of Hertfordshire advises assessing infants’ mucous membranes. Normal mucous membranes appear pink, while blue mucous membranes are considered abnormal and require action [23]. HEE MIST e-learning states that assessing an infant’s oxygenation should not solely rely on their skin colour, and that colour changes in the infant’s tongue, gums, or lips as the most reliable indicator of oxygenation.

The Newborn and Infant Physical Examination (NIPE) is conducted “within the first 72 hours*”* to assess infant wellness [24]. The NIPE screening guideline directs healthcare professionals to monitor neonates’ *“general tone & central and peripheral colour”* during the assessment of potential congenital heart abnormalities, along with observing for any indications of concern, such as:

*“Episodes of apnoea lasting longer than 20 seconds or associated with colour change”* [24] or *“central cyanosis.”* [24, 29] or *“poor colour”.* [29]

Furthermore, healthcare professionals should ask parents if they have seen any changes in their infant’s skin colour when conducting infant screening for heart problems [24, 29].

Within all the policies for routine neonatal care and skin colour assessment, there is no mention of how ethnicity may impact the assessment of skin colour.

### Apgar score

Two guidelines reviewed offered information on conducting Apgar scoring [23, 30]. The training resource [29] noted that the Apgar score should be conducted. The first guideline suggested routinely documenting the Apgar score at 1 and 5 min, although it was only used to guide the participants, without instructions on determining the score [30]. The University of Hertfordshire’s guidelines (referred to by the Neonatal Nurse Association) outline the colour assessment process for determining the Apgar score as follows:

“*White for a score of 0, Blue for a score of 1 and Pink centrally for a score of 2”.* [23].

This finding contrasts with the *conventional* definition of the Apgar score, where a score of 0 corresponds to a *“pale or blue”* appearance, a score of 1 indicates a body that is *“pink with blue extremities”*, and a score of 2 signifies being *“completely pink*” in appearance [31].

The guidelines and training resources [23, 29, 30] did not address ethnicity or potential variations in skin colour assessment for the Apgar score. Nonetheless, the HEE [29] training recognised that colour assessment was considered “*subjective*” and emphasised that it “*should not be relied upon in isolation.”*

### Jaundice

Jaundice detection was examined in seven guidelines [19, 20, 23, 28, 31, 32, 34] and covered in the HEE training resource [29]. The guidelines state that the primary manifestation of jaundice in newborns is a *“yellow”* hue in the skin [28, 29, 31, 34, 35, 38]. , alongside yellowing of the sclerae [29, 31, 34, 35] and the palate or mucous membranes [29, 32]. The description of jaundice as *“yellowing of the skin or whites of the eyes”* was also detailed in the illustrative guide intended for parents of late preterm infants [19].

Further guidelines propose assessing the extremities for yellow discolouration, expressing particular concern when *“yellow palms and soles”* are seen at any age [28, 33]. During the assessment of jaundice, the significance of adequate lighting was emphasised, as jaundice may seem more accentuated in artificial light or at risk of being overlooked in inadequate lighting [28, 32, 35, 38]. Additionally, the Institute of Health visitors provided advice about the detection of prolonged jaundice using the Children’s Liver Disease Foundation stool and urine colour chart [39], with immediate referral to a paediatrician mandated if the stool or urine was abnormal in colour [34].

Multiple guidelines on neonatal assessment recommend regular skin evaluation for signs of jaundice during every interaction [19, 20, 23], particularly in the first 72 h [20, 29]. BAPM [20] stresses checking bilirubin levels if jaundice arises but cautioned against transcutaneous bilirubinometer use within 24 h after birth. HEE [29] encourages the use of transcutaneous bilirubinometers or alternative laboratory assessments when visible jaundice is present and advises healthcare professionals not to *“guess”* jaundice levels solely based on the intensity of yellow discolouration. One guideline [20] highlighted an increased jaundice risk for infants of Asian ethnicity. No guidelines, except for NICE, have addressed how jaundice might manifest in neonates of different ethnicities or supplied strategies to address challenges linked to differences in skin pigmentation and phenotypes. The NICE guidelines and quality standards [32, 35, 38] outline potential diagnostic difficulties in neonates with different skin tones.

Consequently, the NCC-WCH & RCOG guideline (2010) and quality standards [38] state that examining the *“sclerae, gums, and blanched skin is beneficial across all skin tones.”* A later update [35] removed the term *“all skin tones”* and introduced guidance on evaluating *“blanched skin.”*

HHE e-learning (2022) addressed ethnicity by featuring a case study involving a Pakistani infant and prompting participants to identify the infant’s *“risk factors”.* They highlighted that jaundice becomes apparent at approximately 80 µmol/L in infants with *“pale skin”* and suggested that infants from ethnic minority groups might need “closer monitoring”. For darker skin tones, the module recommended that *“assessment of the sclera and mucus membranes may be more reliable”* than relying on skin colour assessment [29].

The NICE guidance recognises that using visual assessment alone for jaundice is not advisable.

However, they also advised against routinely conducting bilirubin measurements in all infants [32, 35]:

In contrast, the latest WHO postnatal care guidance [33] advocates for the universal screening of neonatal hyperbilirubinemia using a transcutaneous bilirubinometer (TCB) upon discharge from health facilities. While recognising total serum bilirubin as the most accurate method of estimation, it is also acknowledged that a heel prick test requires access to a laboratory assessment, which is not globally available. The WHO guidelines concluded that there is insufficient evidence both for and against universal serum bilirubin screening at health facility discharge.

The policies for assessing jaundice predominantly rely on skin colour. Some guidance implies that ethnicity may pose a challenge in detecting jaundice. One specific guideline [33] outlined the potential for transcutaneous bilirubinometer assessments to overestimate bilirubin levels in newborns with darker skin colours, although the existing evidence contradicts this.

### Cyanosis or hypoxia

The identification of cyanosis and/or hypoxia was performed according to eight guidelines/policies [21, 22, 24, 26, 27, 30, 36, 37] and one training resource [29].

In cases where an infant is considered at higher risk (e.g., through passage of meconium in utero or prolonged rupture of membranes), the NICE intrapartum guidelines recommend monitoring for central cyanosis and advise *“confirming by pulse oximetry if available”* [30]. Similarly, a sign considered suggestive of congenital heart disease in neonates is *“central cyanosis”* [24].

When monitoring for respiratory issues, it is recommended to observe *“skin colour, pallor, mottling, cyanosis, and any traumatic petechiae around the eyelids, face, and neck”* [26]. The training resource suggests that an infant displaying a *“mottled,” “blue,”* or *“pale”* colouration may indicate potential respiratory distress [29]. Another guideline highlights “*cyanosis*” as an abnormal respiratory assessment criterion for a neonate [36].

One guideline specifically addressed the assessment of cyanosis in neonates [22]. Skin colour assessment was also performed, prompting healthcare professionals to observe for the neonate *“appearing pale, ashen, mottled, or blue”* [22]. None of these guidelines explicitly describe how cyanosis or central cyanosis may manifest across different skin tones, phenotypes, or ethnic characteristics. However, online resources [29] provide more explicit guidance, suggesting that *“any change from the established centrally pink colour in a newborn is always abnormal”* and that central colour change is most *“reliably noted in the lips, gums, and tongue”* and can present as *“paleness or dusky blue.”*

Another guideline explicitly emphasised that the recognition of cyanosis should not rely solely on skin colour [27].

The training resource [29] went one step further, proposing that *“skin colour also varies according to genetic determinants and therefore is not a good means of assessing oxygenation”.* For this reason, they suggested that any baby with a *“dusky appearance”* should have their pulse oximetry checked [29].

Regarding hypoxia, one guideline emphasised that if an infant appears floppy, has inadequate breathing, and has a very slow heart rate of less than 60 beats per minute, along with “pale” colouration, it suggests significant hypoxia [27]. However, once more, relying solely on visual observation should be avoided:

Therefore, the resuscitation council proposed that in the *“rare circumstance”* of an absence of *“both a pulse oximeter and an air/oxygen blender”*, 100% oxygenation be given and assessed by monitoring the heart rate and colour; however, this should be avoided whenever possible or “*rectified as soon as possible”* [37].

The challenge of evaluating hypoxia in preterm neonates was specifically highlighted in particular guidelines [21]. Currently, there is insufficient evidence of the accuracy of pulse oximetry or transcutaneous measurement of the partial pressure of arterial oxygen compared to arterial oxygen levels [21].

None of the guidelines or training resources covering hypoxia or cyanosis in neonates explicitly stated issues with skin colour assessment or accuracy issues when assessing oxygen saturation in neonates of different ethnicities or skin pigmentations.

### Evidence used in guideline development

Some guidelines, frameworks, and quality standards provide comprehensive evidence supporting the basis of the recommendations. In instances where the recommendations within the guidelines, policies, and frameworks are accompanied by evidence, the rationale is elaborated upon below.

### Jaundice

#### Summary of the NICE evidence

The NICE guidelines [32, 35, 38] evaluate evidence suggesting that darker skin colour can hinder the assessment of neonatal jaundice. The evidence provided in the evidence summary of the 2010 document suggested that the correlation coefficient was much lower for preterm babies and babies with dark skin tones than for babies with light skin tones and term babies [32]. The guideline development group also recognised that in one study, parental assessment of jaundice was more accurate than that of healthcare professionals [40].

The guidelines recognise the challenge of gauging jaundice through visual assessment methods, indicating a *“moderate correlation”* with accuracy. However, they emphasised that bilirubin levels should not be routinely measured in *“babies who are not visibly jaundiced”.*

The data was considered sufficient to demonstrate the precision of transcutaneous bilirubinometers, specifically the Minolta JM-103 and BiliChek, in term infants with low bilirubin levels (bilirubin < 250 µmol/litre) [32]. Additionally, it was suggested that the BiliChek yielded more accurate results than the Minolta JM-102 or JM-103 in individuals with darker skin tones [32]. However, despite being more accurate than visual inspection, the Bilichek was observed to be less precise in individuals with darker skin tones [32]. Nevertheless, the guidelines emphasise the need for further research into *“transcutaneous bilirubin screening”* and associated *“risk factors,”* such as *“dark skin tones,”* to evaluate the diagnostic accuracy of serum bilirubin and transcutaneous bilirubin devices for infants [32]. Furthermore, they noted a dearth of high-quality evidence concerning the use of icterometers in infants with dark skin [32].

### WHO recommendations

The revised WHO guidelines for postnatal care recognise the potential challenges of transcutaneous bilirubin measurements overestimating serum bilirubin levels in newborns with darker skin tones. However, conflicting evidence on this matter has been highlighted [33]. Despite suggesting that transcutaneous bilirubin assessment should be universally conducted upon discharge, the recommendations note the current lack of adequate evidence to support the widespread screening of total serum bilirubin at health facility discharge. This hesitancy was attributed to the substantial associated costs and the considerable variability in the feasibility and acceptability of the test [33].

### Postnatal care: NICE evidence summary

The review and drafting of recommendations in 2021 were undertaken entirely “*afresh”*. In contrast to the 2006 guideline, which stated, *“Healthy babies should have normal colour for their ethnicity”* [41], the more recent guidance from 2021 did not specifically address the varied appearance of healthy skin colour in different ethnicities [22]. In the latest review, all studies related to scoring systems for infant illness and indications of serious illness in infants were deemed to be of very low to moderate quality and lacked substantial evidence. Notably, ethnicity was not a focal point in any of the studies presented in either summary.

### Specialist neonatal respiratory care for babies born preterm: NICE evidence summary

While formulating the NICE guidance on *“specialist neonatal respiratory care for babies born preterm”* in 2021, there were no identified studies examining the diagnostic accuracy of pulse oxygen saturation or colour assessment for neonates [21].

## Discussion

Current UK policy, guidelines and training resources regarding neonatal testing do not appropriately address differences between different skin pigmentation conditions; however, for many neonatal assessments, observations of an infant’s skin colour are still mentioned as predictors of certain illnesses. Neonates were referred to as having certain colours or shades (*“pink”, “blue”, “pale” or “pallor”*), with no differences in terminology used for infants of Black, Asian and ethnic minority backgrounds. An implication here is the possibility that assumptions made by skin colour assessment are relevant only to those with white skin colour and may not be inclusive or representative of the diverse communities that make up our multiethnic society.

The subjectivity of skin colour assessment was recognised within three policies and one training resource, which indicated that skin colour assessment should not be used in isolation to assess neonates. However, for some conditions, a visual assessment by healthcare professionals is still needed before further testing, which may disadvantage Black, Asian and ethnic minorities [8, 42].

Very little information was found in the literature on whether skin colour and the assessment of Black, Asian and ethnic minorities are adequately considered in policy formulation. Recently, attention has been given to the impact of adult skin pigmentation and the diagnosis of certain skin conditions [12, 13]; however, additional research is needed on neonatal demographics, where assessing skin colour is still standard practice.

The jaundice guidelines from NICE [32, 38] and BAPM [20], as well as the training resource from HEE [29], state that serum or transcutaneous bilirubin should be tested if jaundice is *“visible”* or there are concerns about jaundice and that bilirubin levels should not be estimated from visual assessment alone. Within the guidance, healthcare professionals were advised that bilirubin levels should not be measured routinely, as visual inspection should be performed initially [29, 32, 38], which may disadvantage those with darker skin pigmentation. Visual inspection of jaundice has repeatedly been found to be unreliable [8, 43], with instances of jaundice being missed by healthcare professionals, notably in those with darker skin pigmentation [44]. Indeed, the NICE guidance deemed the detection of jaundice in darker skin tones to be *“almost impossible”* [32]. The issue of jaundice going undetected in darker-skinned infants is of particular importance, as guidelines have identified infants of Asian ethnicity to be at increased risk of jaundice [20]. Hence, it could be hypothesised that those at the highest risk of developing jaundice are the most likely to be missed by means of visual assessment. The consequences of missed jaundice can profoundly impact infants and their families, potentially leading to kernicterus, a condition shown to disproportionately affect non-Caucasian infants [46, 47].

The guidelines indicated that cyanosis be “*observed for”* with the use of descriptors such as *“dusky appearance”* [29] or *“pale, ashen, mottled or blue”* [22]. Resources suggest that if such colours are observed in an infant or if central cyanosis is suspected, pulse oximetry should be performed [29, 30]. However, it is acknowledged that colour descriptors for skin may not be appropriate when assessing cyanosis in ethnic minorities, and new parameters may be required [14]. Policies and training resources did not refer to differences that may be seen in cyanosed infants from an ethnic minority or comment on the relevance of assessing skin colour. Therefore, the deterioration of a Black, Asian or ethnic minority neonate may be identified too late in the absence of a pulse oximeter.

Numerous policies and training resources suggest that colour is not an appropriate assessment tool for assessing oxygenation in isolation [29, 37] and should be used only in the absence of pulse oximetry [37]. However, inequalities highlighted by the COVID-19 pandemic suggested a bias in pulse oximetry readings for those with darker or different skin colours compared to white individuals [14], including among newborns [15]. None of the policies or training resources addressed this potential racial bias in technology or assessment of oxygenation, which is an essential avenue for future research.

Guidelines and training resources suggest observing a neonate for colour change to identify heart abnormalities [24, 29]. However, it is noted that colour change may not be apparent in infants with darker skin pigmentation [11]; therefore, it may remain unobserved by parents and professionals.

Several policies and guidelines provided inadequate descriptions of the evidence base used to produce the policy/guidelines. To develop a full picture of the validity of the policies, the evidence used in their formulation will need to be displayed. The evidence base used to model any guideline should definitively include samples from a wide spectrum of ethnicities and races to ensure the applicability of the guidance to the UK population.

### Implications for policy

All policies and guidelines related to neonatal assessment by skin colour must acknowledge and address potential differences for neonates from black, Asian, and ethnic minority backgrounds. These guidelines must incorporate ethnicity-inclusive descriptive terms and provide specific assessments for different skin pigments and phenotypes. If certain guidelines lack images or accurate descriptors for skin colour assessments across all pigmentations, they should refer to such resources. Additionally, the reliability and accuracy of skin colour assessment as a standalone diagnostic tool should be thoroughly discussed, with special attention given to variations in reliability or accuracy for neonates with diverse skin pigmentation. Professional associations should play a role in identifying and implementing necessary training to ensure full competence in skin colour assessments for infants from these backgrounds. Moreover, the evidence base supporting neonatal assessment guidelines must indicate the ethnicity of participants in all studies to identify gaps in understanding the impact of ethnicity and enhance transparency regarding the relevance of the evidence to all ethnicities.

### Strengths and limitations

The research paper benefits from an extensive policy review encompassing various organisations and sites. In addition, the researchers invited stakeholders to contact the research team if they were aware of any relevant policy or article in line with the study’s objectives. The research was limited to the United Kingdom. By limiting the study to the UK context, this approach allows us to consider the unique policies and practices that are relevant to the UK, providing valuable insights for policymakers, practitioners, and stakeholders operating within this region. Nevertheless, it is crucial to recognise and consider the possible limitations and adverse consequences associated with this approach, especially concerning its applicability and the potential oversight of broader global viewpoints. This research lacks a specific critical appraisal of the reviewed policies and guidelines, as the reviewer felt it was unnecessary for the scope of the review due to the review’s focus on the policy’s consideration of ethnicity.

## Conclusion

This review aimed to assess UK policy and to understand whether ethnicity and race are appropriate considerations in policy formulation regarding skin colour and neonatal examinations. The impact of ethnicity was poorly considered during policy formulation and the development of guidelines and training. These results further perpetuate the inequalities faced by Black, Asian and ethnic minorities by means of improper assessment or potential late diagnosis. Therefore, there is a need for a review of policy, guidelines and training resources and their contributing evidence to ensure that they are relevant to all neonates, regardless of skin pigmentation.

### Electronic supplementary material

Below is the link to the electronic supplementary material.

Supplementary Material 1

Supplementary Material 2

## Data Availability

All data generated or analysed during this study are included in this published article [and its supplementary information files].

## Abbreviations

AGREE II: Appraisal of Guidelines for Research & Evaluation II instrument
BAPM: British Association of Perinatal Medicine
COVID-19: SARS-CoV-2 (Coronavirus)
HEE: Health Education England
iHV: Institute of Health Visiting
NHS: National Health Service
NICE: National Institute for Health and Care Excellence
NIPE: Newborn and Infant Physical Examination
NNA: Neonatal Nurses Association
RCM: Royal College of Midwives
RCN: Royal College of Nursing
RCOG: Royal College of Obstetricians and Gynaecologists
RCPCH: Royal College of Paediatrics and Child Health
TCB: Transcutaneous bilirubin level
UK: United Kingdom
WHO: World Health Organization
