# Supplementary material for: A review of the current policies and guidance regarding Apgar scoring and the detection of jaundice and cyanosis concerning Black, Asian and ethnic minority neonates
Source: BMC Pediatr. 2024 Mar 21;24:198. doi: 10.1186/s12887-024-04692-4 (PMC10956215; doi:10.1186/s12887-024-04692-4)
Supplement: Supplementary file 1 — Supplementary Material 1 [file 12887_2024_4692_MOESM1_ESM.docx]

**Additional file 2:**

**Routine care**

General skin colour:

*“Colour, tone, breathing and heart rate”* (25) or

*“Colour, activity, temperature, heart rate and respiratory rate for the first 24 hours.”* (19)

*A “normal baby should be centrally pink, though the extremities of hands and feet are usually tinged blue”.* (29)

After the neonate has detached from the breast, it should be assessed to ensure that it is *“breathing well, pink and warm”* (28).

Discoloured peripheries and alternative locations:

*“The baby should be assessed by looking at the whole of the baby’s body, as the limbs can often be discoloured first. Subtle changes to colour indicate changes in the baby’s condition.”* (20)

*“Episodes of apnoea lasting longer than 20 seconds or associated with colour change”* (24), or *“central cyanosis”* (29,24), or *“poor colour”.* (29)

*“Parents should be asked if their baby ever gets breathless or changes colour at rest or while feeding”* (24).

**Apgar score**

“*White for a score of 0, Blue for a score of 1 and Pink centrally for a score of 2”.* (23).

**Jaundice**

*“Detection of jaundice in babies with dark skin tones can be almost impossible”* (32).

*“Babies with very pale skin can appear “suntanned” rather than yellow.”* (32).

*“Clinical recognition and assessment of jaundice can be difficult, particularly in babies with darker skin tones.”* (35).

*“Do not rely on visual inspection alone to estimate the bilirubin level in a baby with suspected jaundice”* (32,38,35).

*“Examine the sclerae and gums and press lightly on the skin to check for signs of jaundice in ‘blanched’ skin.”* (35)

*“Do not rely on visual inspection alone to estimate the bilirubin level in a baby with suspected jaundice”* (32,38,35).

“*Do not measure bilirubin levels routinely in babies who are not visibly jaundiced”* (32,35).

**Cyanosis or hypoxia**

*“Colour is a poor means of judging oxygenation, as cyanosis can be difficult to recognise”* (27).

*“One cannot accurately assess oxygenation, and particularly hyperoxia, by colour alone”* (37).
