# Supplementary material for: A review of the current policies and guidance regarding Apgar scoring and the detection of jaundice and cyanosis concerning Black, Asian and ethnic minority neonates
Source: BMC Pediatr. 2024 Mar 21;24:198. doi: 10.1186/s12887-024-04692-4 (PMC10956215; doi:10.1186/s12887-024-04692-4)
Supplement: Supplementary file 2 — Supplementary Material 2 [file 12887_2024_4692_MOESM2_ESM.docx]

**Additional file 1. Search Terms used within the different professional and organisational sites.**

**BAPM**

There was no facility to search on the BAPM website therefore the ‘Resources’ tab was searched manually for relevant documents.

**Cochrane Library database**

"Neonatal Guidelines" "cyanosis guidelines" "hypoxia guidelines" "Skin Colour" "Skin Color" "Jaundice Guidelines " "pulse oximetry" "oxygen saturation"

Searched then filtered by “Child health” then subsequently by “Neonatal care”

**DANS Easy**

"Neonatal Guidelines" "cyanosis guidelines" "hypoxia guidelines" "Skin Colour" "Skin Color" "Jaundice Guidelines " "pulse oximetry" "oxygen saturation"

**Google**

“Neonatal jaundice AND UK guidelines OR policy” “Hyperbilirubinemia AND UK guidelines OR policy” “Neonatal cyanosis AND UK guidelines OR policy” “Neonatal hypoxia AND UK guidelines OR policy” “APGAR score AND different skin colours” “APGAR score AND Black infants” “Apgar score AND Asian infants” “Neonatal OR Newborn care AND UK guidelines OR policy” “Apgar score AND UK guidelines OR policy” “Neonatal OR Newborn skin colour AND policy OR guidelines” “Neonatal OR Newborn oxygen saturation AND guidelines OR policy” “Neonatal OR Newborn pulse oximetry AND guidelines OR policy” “Neonatal Jaundice AND different skin colours” “Cyanosis AND different skin colours” “Hypoxia AND different skin colours”

The first 100 results were reviewed at the title. It is important to note that Google search results may vary based on users' cookie settings and browsing history.

**Institute of Health Visitors**

"Neonatal" "Neonate" "cyanosis" "hypoxia" "Asian" "Black" "Colour" "Color" "Jaundice" "pulse oximetry" "oxygen saturation"

**Neonatal Nurses Association**

No search terms. The ‘Learn’ tab was screened including the links to additional resources which led to “Neonatal nursing – knowledge to support learning in practice” resources:

<https://www.herts.ac.uk/study/schools-of-study/health-and-social-work/course-subject-areas/nursing/childrens-nursing/neonatal-nursing>

**NICE**

"Neonatal" "Neonate" "cyanosis" "hypoxia" "Asian" "Black" "Colour" "Color" "Jaundice" "pulse oximetry" "oxygen saturation"

**Office for Health Improvement & Disparities.**

Filtered by ‘guidance and regulation’

“Black Neonate” “Asian Neonate”

"Neonatal" "Neonate" "cyanosis" "hypoxia" "Asian" "Black" "Colour" "Color" "Jaundice" "pulse oximetry" "oxygen saturation"

**Resuscitation council**

"Neonatal" "Neonate" "cyanosis" "hypoxia" "Asian" "Black" "Colour" "Color"

**Royal College of Obstetricians and Gynaecologists**

“Apgar" "Neonatal" "cyanosis" "hypoxia" "Asian" "Black" "Colour" "Color"

**Royal College of Midwives**

"Apgar" "Neonatal" "cyanosis" "hypoxia" "Asian" "Black" "Colour" "Color"

**Royal College of Nursing**

"Neonatal" "Neonate" "cyanosis" "hypoxia" "Asian" "Black" "Colour" "Color" "Jaundice" "pulse oximetry" "oxygen saturation"

**Royal College of Paediatrics and Child Health**

**Filtered by content type: ‘Clinical guidelines and Standards’**

"Neonatal" "Neonate" "cyanosis" "hypoxia" "Asian" "Black" "Colour" "Color" "Jaundice" "pulse oximetry" "oxygen saturation"

**WHO**

"Neonatal" "Neonate" "cyanosis" "hypoxia" "Asian" "Black" "Colour" "Color" "Jaundice" "pulse oximetry" "oxygen saturation"
